# Lung Involvement in Children with Hereditary Autoinflammatory Disorders

**DOI:** 10.3390/ijms17122111

**Published:** 2016-12-15

**Authors:** Giusyda Tarantino, Susanna Esposito, Laura Andreozzi, Benedetta Bracci, Francesca D’Errico, Donato Rigante

**Affiliations:** 1 Institute of Pediatrics, Università Cattolica Sacro Cuore, Fondazione Policlinico Universitario Agostino Gemelli, 00168 Rome, Italy; giusyda16@hotmail.it (G.T.); laurandreozzi@gmail.com (L.A.); benedetta.bracci@gmail.com (B.B.); francscaderrico1403@gmail.com (F.D.); 2 Pediatric Highly Intensive Care Unit, Department of Pathophysiology and Transplantation, Università degli Studi di Milano, Fondazione IRCCS Ca’ Granda Ospedale Maggiore Policlinico, 20122 Milan, Italy; susanna.esposito@unimi.it

**Keywords:** autoinflammatory disorder, pleuritis, interstitial lung disease, child

## Abstract

Short-lived systemic inflammatory reactions arising from disrupted rules in the innate immune system are the operating platforms of hereditary autoinflammatory disorders (HAIDs). Multiple organs may be involved and aseptic inflammation leading to disease-specific phenotypes defines most HAIDs. Lungs are infrequently involved in children with HAIDs: the most common pulmonary manifestation is pleuritis in familial Mediterranean fever (FMF) and tumor necrosis factor receptor-associated periodic syndrome (TRAPS), respectively caused by mutations in the *MEFV* and *TNFRSF1A* genes, while interstitial lung disease can be observed in STING-associated vasculopathy with onset in infancy (SAVI), caused by mutations in the *TMEM173* gene. The specific pleuropulmonary diseases may range from sub-clinical abnormalities during inflammatory flares of FMF and TRAPS to a severe life-threatening disorder in children with SAVI.

## 1. Introduction

Hereditary autoinflammatory disorders (HAIDs) are defined by seemingly unprovoked self-limited febrile attacks combined with signs of systemic inflammation recurring in different organs and tissues, as a direct consequence of dysregulated innate immune pathways and without a clear evidence of adaptive immune dysfuntion, high-titer autoantibodies or antigen-specific T cells [1,2]. Patients with HAIDs display quite prototypical clinical characteristics and present symptom-free intervals of variable duration between attacks, which usually start in the pediatric age [3]. The unifying pathogenetic mechanism of HAIDs is represented by abnormal interleukin (IL)-1 signaling and delayed shutdown of a normal inflammatory response [4]. As a first distinction, all autoinflammatory syndromes can be divided in monogenic HAIDs (listed in Table 1) and multifactorial polygenic disorders, including Behçet’s syndrome, adult-onset Still’s disease, systemic-onset juvenile idiopathic arthritis, recurrent pericarditis, and PFAPA (periodic fever, aphthous stomatitis, pharyngitis, cervical adenitis) syndrome: the diagnostic identification of one among these conditions derives from the integration of familiar, clinical, biohumoral, and genotype investigations [5,6].

The goal of this review is to analyze the occurrence of inflammatory signs in the respiratory system during the typical attacks of patients with HAIDs: on PubMed, we searched for all eligible studies published over the last 15 years, matching the keywords “lung” or “pulmonary” and “autoinflammation”, but due to the low number of articles retrieved we matched specifically “lung” or “pulmonary” with the names of each single HAID.

## 2. Familial Mediterranean Fever

Familial Mediterranean fever (FMF) was initially described in 1945 as “benign paroxystic peritonitis” in patients displaying recurrent peritonitis and periodic short fever attacks: this is the most common among all HAIDs and the first one with the causing gene identified in 1997, named *MEFV*, which encodes a 781-amino acid protein, wherever known as pyrin [7]. At present, 314 *MEFV* sequence variants have been associated with FMF (http://fmf.igh.cnrs.fr/infevers). The disease is characterized by recurrent self-resolving attacks of fever, abdominal, thoracic or joint pain and variable degrees of systemic inflammation with intercritical period of apparent wellness. Onset symptoms begin in about 50% of cases during the first decade and in some cases even during the first year of life. Features of a typical attack include fever and the potential occurrence of either serositis or arthritis, lasting from 1 to 3 days, resolving spontaneously, but recurring with irregular periodism [8]. Abdominal pain is present in about 90% of patients and is the predominant clinical manifestation in half of them, with a clinical scenery simulating acute abdomen, as in appendicitis, cholecystitis, or urolithiasis [9].

Chest-related manifestations are less frequent in FMF, and pleurisy is the most common cause: a primary attack of pleuritic chest pain associated with high-peaking fever has been described in 10% of pediatic patients, but approximately 30%–40% may manifest an attack of febrile pleurisy during the natural course of an untreated FMF, lasting less than 4 days and resolving without treatment. Physical examination may be nonspecific in young patients and is characterized by unilateral chest pain that increases in inspiration, shortness of breath, and rapid shallow breathing.

Chest imaging findings are usually nondiagnostic, although occasionally the costo-phrenic angle may be blunted on the side of the attack, while recurrent attacks may sometimes cause pleural thickening and/or adhesions. The pleural fluid, if aspirated, might reveal a prevalent rate of neutrophils [10].

The first case of FMF presenting with recurrent pulmonary atelectasis, which was responsive to continuous colchicine therapy, was described in 1987 by Brauman & Gilboa in a young man of Jewish-Georgian ancestry [11]. Pleural inflammation may often result in a misdiagnosis of pneumonia, sometimes deriving from atelectasis that accompanies pleurisy; if chest attacks are the first or, more rarely, the only manifestation in children, the final diagnosis of FMF may even be delayed for years, and the young patient may receive unnecessary antimicrobial therapies with a needless risk of toxic effects on one hand and a more relevant risk of unchecked amyloidogenesis on the other, as colchicine prophilaxys is not started [12].

In 2003, Brik et al. documented lung-related manifestations in 48 genetically-diagnosed FMF patients, aged 6–18 years: all of them underwent complete pulmonary function tests, including spirometry, total lung capacity via body plethysmography, and single-breath determination of diffusing capacity of the lung for carbon monoxide. It was shown that pulmonary symptoms and/or signs (such as chest pain, shallow breathing, cough) during FMF attacks were significantly more common (40%) in Jewish patients carrying in homozygosis the M694V *MEFV* mutation than in Arabians homozygotes for the V726V mutation; a mild restrictive lung disease was also found in a small number of patients homozygotes for the M694V mutation, who also presented a particularly severe disease course (only 3, i.e., 6% of the total cohort). Although this series was small to draw definite conclusions about long-term consequences in the respiratory system of FMF patients, the rate of pulmonary symptoms was higher than in the healthy pediatric population, and this could probably be explained by the typical recurrence and sum of occult parenchymal injuries during recurrent acute attacks [13].

In 2008, Katsenos et al. reported the first case of FMF-related lymphocytic exudative unilateral pleuritis, which recurred in a three-month-period in combination with low-grade fever, in a 30-year-old man of Greek origin, which led to the diagnosis of FMF and was successfully treated with continuous colchicine administration [14]. No cases of lymphocitic effusion have been observed in children or adolescents with a confirmed FMF.

Pulmonary infiltrates resulting from other FMF-related conditions, particularly vasculitides or thromboembolism, may occur in untreated or noncompliant patients; therefore, they should be considered in the differential diagnosis [15]. Isolated vasculitis of the lung is possible, but only one has been reported—namely, a case of isolated pulmonary vasculitis in which FMF had started at 17 years, showing a lung infarction and loss of 40% of the original number of respiratory units in the lung as a consequence of occlusion of pulmonary capillaries, was reported in 1971 [16].

Amyloidosis of the lung is a rare dreadful complication of untreated FMF, mostly associated with symptomatic involvement of other organs, such as the kidney: diffuse pulmonary amyloidosis secondary to FMF, although infrequent, may cause clinical and radiologic findings that mimic a chronic interstitial lung disease, definitely related to the amyloid protein deposition in the vessel walls or in the alveolar septa [17]. Clinicians should be aware that granulomatous nodules and ground-glass opacities may be additional pulmonary manifestations in patients with FMF, though they have only been reported in one adult patient [18].

An attractive theory explaining the high prevalence of FMF heterozygotes in the Mediterranean Sea is that patients or asymptomatic carriers display a selective advantage in relationship with the risk of asthma. Danon et al., in 1990, using the Israeli army database, demonstrated for the first time a significantly lower prevalence of asthma in FMF patients compared to the general prevalence [19]. Furthermore, another study by Ozyilkan et al. confirmed the same finding in a group of 100 FMF patients without asthma [20], and, in addition, Brenner-Ullman et al. found an apparently reduced prevalence of asthma in the FMF heterozygotes compared with controls [21]. Although the question of protection against asthma in FMF remains open, the possibility of a negative association between the two diseases is appealing and requires further confirmation studies.

## 3. Tumor Necrosis Factor Receptor-Associated Periodic Syndrome

Tumor necrosis factor receptor-associated periodic syndrome (TRAPS) is the most frequent autosomal dominant autoinflammatory disorder, caused by mutations in the *TNFRSF1A* gene, encoding the tumor necrosis factor (TNF)-receptor type 1 [22]. At present, 147 *TNFRSF1A* sequence variants have been associated with TRAPS (http://fmf.igh.cnrs.fr/infevers). The disease was initially described in 1982 as “familial Hibernian fever”, reflecting the Irish and Scottish ancestry of patients in the very first reports, and is characterized by longlasting febrile attacks variously accompanied by abdominal pain, migratory rashes, musculo-skeletal symptoms, and ocular signs, which can recur many times a year [23]. All patients, both children and adults, present a significant heterogeneity of clinical features, probably due to the broad spectrum of *TNFRSF1A* mutations: the average age at disease onset is around three years, and some of these children are frequently misdiagnosed with recurrent infections of childhood [24].

Serous membrane inflammation is commonly observed in TRAPS patients, usually in the form of polyserositis, as well as in FMF [25]. Chest pain can be caused by either aseptic pleural inflammation, or elective involvement of thoracic muscles: the overall duration of symptoms of polyserositis may vary from a few days to several weeks, much longer than that in FMF, usually accompanied by high-grade fever and resolving without any specific treatment [26]. Pleuritic chest pain was described for the first time in 40% of children with a genetically confirmed diagnosis of TRAPS, recruited by the National Institute of Health Clinical Centre in Bethesda [27]. Pelagatti et al. reported the clinical features of 11 Italian patients with TRAPS, aiming to analyze the long-term impact of the R92Q *TNFRSF1A* mutation in these children, who displayed a lower frequency of typical TRAPS manifestations, and they noted that recurrent febrile thoracic pain/pleuritis occurred in 27.2% of cases [28]. Chest pain lasting for more than two weeks “without” fever has also been reported in TRAPS, showing that systemic inflammation might be dissociated by fever in this condition [29].

Lachmann et al. have evaluated the demographic, clinical, and genetic findings of TRAPS patients recruited from an international registry of periodic fever syndromes: 34% of them were children or adolescents aged less than 18 years, of both sexes, with a median age of 1.5 years at disease onset and a mean diagnostic delay of 2.7 years; although disease features seemed similar between children and adults at presentation, recurrent chest pain, and acute pleurisy were less frequent in childhood than in the adult population (20% versus 54%), and mainly occurred in young patients with the T50M *TNFRSF1A* variant [30].

Most TRAPS cases have been diagnosed in patients of European ancestry, but a set of nine young patients with TRAPS has been recently reported in Japan: only a minority of them showed recurrent chest pain (22.2%), conjunctivitis, and/or headache, suggesting a milder disease course than in the larger group of Caucasian patients [31]. In 2006, Manki et al. described two Japanese siblings with TRAPS (caused by the missense C30Y *TNFRSF1A* mutation in both) who presented pleural involvement and a characteristic decrease of serum soluble TNF-receptor, initially misdiagnosed as systemic-onset juvenile idiopathic arthritis at onset, suggesting that TRAPS can be frequently overlooked or misdiagnosed, and that TRAPS should be considered in all pediatric patients with atypical recurrent inflammatory manifestations [32].

## 4. STING-Associated Vasculopathy with Onset in Infancy

A peculiar and severe syndrome among HAIDs which occurs in very young children is STING (stimulator of interferon genes)-associated vasculopathy with onset in infancy, named SAVI and caused by gain-of-function mutations in the *TMEM173* gene, which encodes the STING protein: STING activation is followed by constitutionally increased interferon-β transcription and a typical “interferon signature” in the blood [33].

SAVI is considered part of a growing group of Mendelian disorders defined “interferonopathies”, which include Aicardi-Goutiéres syndrome and chronic atypical neutrophilic dermatosis with lipodystrophy and elevated temperature (CANDLE) syndrome, all characterized by severe uncontrolled activation of interferon and downstream genes. At present, four *TMEM173* sequence variants have been associated with SAVI (http://fmf.igh.cnrs.fr/infevers) [34]. Patients with SAVI have severe neonatal-onset small vessel vasculitis, which is expressed by telangiectatic ulcerative rashes in the limbs, earlobes, or nose, leading to microangiopathic thrombosis, vessel occlusion, and even risk of gangrene.

Some SAVI patients may present chronic interstitial lung disease, which can be severe and lethal [35]. In fact, the STING protein is expressed not exclusively in the vascular endothelial cells, but also in alveolar type II pneumocytes and in bronchial epithelium and alveolar macrophages, explaining the specific lung pathology: STING-induced dysfunction results in a vaso-occlusive process with activation of both local macrophages and pneumocytes [36]. Pulmonary disease appears mostly after skin manifestations and range from mild symptoms, such as recurrent wheezing, to progressive moderate-to-severe dyspnea. Chest radiographs usually show increased interstitial and peribronchial thickening throughout both lungs, while diffuse reticular opacities, lung hyperinflation, air trapping, honeycombing, lymphadenopathy, and traction bronchiectasis might appear at the high-resolution computed tomographic scan. Bronchoalveolar lavage, when done, reveals an inflammatory infiltrate with a large amount of lymphocytes, and lung biopsy is typically characterized by type II pneumocyte hyperplasia, lymphoid infiltration, and interstitial fibrosis [37]. As this respiratory component may predominate over time and have a causative role in mortality, early onset of fever combined with interstitial lung disease starting within the first eight weeks of life, persistent rash, and failure to thrive should definitely suggest the diagnosis of SAVI [38].

Munoz et al. reported on a child where SAVI started at 2 months: the association between skin lesions and interstitial lung disease with positive anti-neutrophil cytoplasmic antibodies (not typed) led the clinicians to an erroneous diagnosis of childhood-onset granulomatosis with polyangiitis (Wegener granulomatosis) at 3–5 months; pulmonary involvement was consistent with the previously described cases of SAVI, but differed in the absence of paratracheal adenopathy. Of interest, an improvement of interstitial lung disease was obtained after treatment with methylprednisolone pulses and mycophenolate mofetil, although this treatment failed to halt the progression of skin lesions over time to violaceous scaling and atrophic plaques on the toes of both hands and feet, with nail dystrophy; nasal septum perforation occurred at 6 years of age, though his pulmonary lesions remained stable from a radiologic perspective [39].

Picard et al. reported three young patients with SAVI, two familial and one sporadic, all displaying pulmonary manifestations suggestive of lung fibrosis at disease onset: the disease was resistant to corticosteroids, and lung transplantation was considered at an early age [40]. As chronic interstitial lung disease in SAVI might be fatal, there is an urgent need for effective therapies in this condition: a clinical trial with baricitinib, a Janus kinase inhibitor that blocks the interferon signaling cascade, is currently in progress and recruiting patients (www.clinicaltrials.gov NCT01724580).

## 5. Other Hereditary Autoinflammatory Disorders

Data on the prevalence and type of pulmonary involvement in children with HAIDs different from FMF, TRAPS, and SAVI are quite scarce. Lung injury may be caused by the disease itself or by the use of selected biologic or non-biologic drugs (as methotrexate) in a few cases. Interstitial lung disease has rarely been reported in Blau syndrome, a familial granulomatous disorder caused by mutations in the *CARD15/NOD2* gene, encoding the cytosolic NOD2 protein (nucleotide-binding oligomerization domain protein 2), one of the key molecules in the regulation of innate immunity: the disease starts in the first years of life with articular, cutaneous, and ocular non-caseating granulomatous inflammation [41]. This type of inflammation rarely affects the lungs or intrathoracic lymph nodes in Blau syndrome, and only one pediatric case with the R334Q *CARD15/NOD2* mutation has been associated with interstitial pneumonia [42].

In addition, autosomal dominant gain-of-function mutations in the *PLCG2* gene cause PLCγ2-associated antibody deficiency and immune dysregulation (PLAID), also named “familial cold autoinflammatory syndrome 3” (FCAS3), clinically characterized by cold-induced urticaria, autoimmune manifestations, and susceptibility to infections. A variant of this specific disorder is named “autoinflammation and PLAID” and may be associated with heterogeneous skin lesions, arthralgias, corneal erosions, and interstitial pneumonia [43].

Lastly, COPA syndrome results from autosomal dominant mutations affecting a narrow amino acid stretch in the *COPA* (coatomer subunit α) gene, encoding the COPα protein, involved in transiting molecular cargo from the Golgi complex to the endoplasmic reticulum: the syndrome, one of the most recently discovered primary immunodeficiencies, which is halfway between autoimmunity and autoinflammation, is typically characterized by the combination of arthritis and interstitial lung disease with a substantial risk of pulmonary hemorrhage. COPA syndrome is also associated with autoantibody production, an increased number of T cells with skewing toward a Th17 phenotype, aberrant cellular autophagy, and oversecretion of different pro-inflammatory cytokines, including IL-1β and IL-6 [44].

## 6. Future Directions in the Evaluation of Lung Involvement in Children with Hereditary Autoinflammatory Disorders

There is a growing scientific interest in HAIDs, as this family of rare heterogeneous syndromes provides information of utmost importance about the intricate pathways of inflammation in general: these last years have witnessed a substantial re-evaluation of many presumably multifactorial diseases, which have been linked to excessive bioactivity of IL-1, as demonstrated by the dramatic response to the available IL-1 inhibitors [45].

Apart from the common phenotype of lifelong recurrent inflammatory attacks, all HAIDs have distinct features (requiring specific therapeutic options), and inflammatory signs may be localized to different target organs or tissues. Moreover, the respiratory system can sometimes be involved in terms of pleural disease in FMF and TRAPS, and in form of chronic interstitial lung disease in SAVI.

Additionally, as HAIDs can be in some rare cases complicated by the occurrence of macrophage activation syndrome (MAS), a potentially fatal complication with high mortality rate, frequently observed in patients with systemic juvenile idiopathic arthritis or many other rheumatologic conditions, infections, and tumors [46], lungs may also be critically involved during the course of HAID-associated MAS, which is driven by dysregulated lymphocyte and macrophage hyperproliferation and continuous overexpression of cytokines [47]. The hallmark of MAS is phagocytosis of blood cells and their precursors: bone marrow aspiration typically reveals the normal maturity of all cell lineages, and infiltration by activated macrophages stuffed with other blood cells [48]. The complex of MAS signs is nonspecific, and none of the biochemical abnormalities can be considered frankly distinctive, though the clinical presentation is usually dramatic, including multiple visceral failure and respiratory distress of variable severity [49]. Pulmonary involvement in patients with MAS can mimic a severe pneumonia or alveolar hemorrhage, and exact differential diagnosis might be challenging: very few patients with FMF, TRAPS, mevalonate kinase deficiency, and cryopyrin-associated periodic syndrome have been reported as showing MAS clinical features thus far [50].

## 7. Conclusions

Respiratory diseases should be carefully assessed in children with HAIDs, and those suspected as having chronic lung involvement strictly evaluated for the risk of pulmonary fibrotic changes. Unfortunately, the availability of biomarkers and chemokines to suggest subclinical pulmonary manifestations in subjects with HAIDs is missing. Moreover, it is unclear whether specific biomarkers of alveolar damage, such as surfactant proteins, or diffuse parenchymal lung damage, such as the glycoprotein KL-6 expressed by type II pneumocytes, measured during inflammatory attacks, might be associated with an adverse prognosis. Furthermore, the lack of histopathological studies of lung parenchyma and pleura in patients with HAIDs displaying respiratory symptoms is an overall limitation in shedding light about this specific kind of organ involvement.

However, the involvement of lungs and airways remains a rare event in HAIDs, varying from simple pleural effusions to chronic fibrosing interstitial lung diseases: this review has suggested a rationale for requiring lung function studies or diagnostic screening tests in young patients with a confirmed autoinflammatory syndrome.

## Figures and Tables

**Table 1:** Genetic characterization of the hereditary autoinflammatory disorders with their pattern of inheritance.

Disease	Gene	Locus	Protein Encoded	Inheritance
Familial Mediterranean fever	*MEFV*	16p13.3	Pyrin	recessive
Tumor necrosis factor receptor-associated periodic syndrome	*TNFRSF1A*	12p13	Tumor necrosis factor receptor type 1	dominant
Mevalonate kinase deficiency	*MVK*	12q24	Mevalonate kinase	recessive
Cryopyrin-associated periodic syndrome	*NLRP3*	1q44	Cryopyrin (NLRP3 protein)	dominant
Interleukin-1 receptor antagonist deficiency	*IL1RN*	2q	Interleukin-1 receptor antagonist	recessive
Majeed syndrome	*LPIN2*	18p11.31	Lipin 2	recessive
Blau syndrome	*NOD2/CARD15*	16q12.1	NOD2/CARD15	dominant
*NLRP12*-associated autoinflammatory disorder	*NLRP12*	19q13.42	Monarch-1	dominant
Proteasome-associated autoinflammatory syndrome	*PSMB8*	6p21.32	Proteasome subunit b type 1	recessive
STING-assocoated vasculopathy with onset in infancy	*TMEM173*	5q31	STING protein	dominant
PAPA syndrome/early-onset sarcoidosis	*PSTPIP1*	15q24.3	CD_2_ antigen-binding protein 1	dominant
Deficiency of adenosine deaminase 2	*CECR1*	22q11.1	Adenosine deaminase 2	recessive
Recurrent hydatidiform mole	*NLRP7*	19q13	NLRP7 protein	recessive

NLRP: NACHT (neuronal apoptosis inhibitor protein, class 2 transcription activator of the MHC, heterokaryon incompatibility and telomerase-associated protein 1), LRR (leucine-rich repeat) and PYD (pyrin domain) domains-containing protein 12; STING: stimulator of interferon genes; PAPA: pyogenic arthritis, pyoderma gangrenosum and acne; NOD2: nucleotide-binding oligomerization domain protein 2; CARD 15: caspase recruitment domain-containing protein 15.
